# *Aristolochia sinoburmanica* (Aristolochiaceae), a new species from north Myanmar

**DOI:** 10.3897/phytokeys.94.21557

**Published:** 2018-01-29

**Authors:** Bin Yang, Hong-Bo Ding, Shi-Shun Zhou, Xinxin Zhu, Ren Li, Mya Bhone Maw, Yun-Hong Tan

**Affiliations:** 1 Southeast Asia Biodiversity Research Institute, Chinese Academy of Sciences, Yezin, Nay Pyi Taw 05282, Myanmar; 2 Center for Integrative Conservation, Xishuangbanna Tropical Botanical Garden, Chinese Academy of Sciences, Menglun, Mengla, Yunnan 666303, P.R. China; 3 College of Life Sciences, Xinyang Normal University, Xinyang, Henan, 464000, P.R. China

**Keywords:** Kachin state, *Aristolochia*, Aristolochiaceae, field expedition, Myanmar

## Abstract

*Aristolochia sinoburmanica* Y.H.Tan & B.Yang, a new species of Aristolochiaceae from Putao, Kachin State, Myanmar, is described and illustrated. According to morphology (strongly curved perianth, 3-lobed limb, as well as 3-lobed gynostemium, anthers 6, adnate in 3 pairs to the base of gynostemium, opposite to the lobes), the species belongs to Aristolochia subgenus Siphisia. It is morphologically similar to *A. faviogonzalezii*, *A. hainanensis*, *A. tonkinensis*, *A. saccata* and *A. xuanlienensis*. The major differences between them are outlined and discussed. A detailed description, along with line drawings, photographs, habitat, distribution and conservation status, as well as a comparison to morphologically similar species, are also provided.

## Introduction

*Aristolochia* L., with about 550 recognised species (González 2012), is a predominantly tropical and subtropical genus that extends to the Mediterranean and temperate zones worldwide and has highest species richness in the New World (Wanke et al. 2006). The genus is also rich in Asia, particularly in eastern and southern Asia, with more than 70 species (Ma 1989; Do et al. 2015a). There are currently 61 species recorded in China (Zhu et al. 2016, 2017), 22 species in Vietnam (Do and Nghiem 2017) and 12 species in Myanmar (Kress et al. 2003). Recent phylogenetic studies of the genus based on morphological and molecular data suggested the subdivision of Aristolochia into three subgenera, i.e. subgenus Aristolochia, subgenus Siphisia and subgenus Pararistolochia (Wanke et al. 2006). The distribution and key morphological characters of the subgenus Siphisia have been described and discussed by González et al. (2014) and Do et al. (2015a, 2015b).

During a field expedition to Putao, Kachin state, north Myanmar, an unknown species of *Aristolochia* was collected. After careful studies of the genus, particularly the floral characteristics of those species in the adjacent regions, as well as comparison between this unknown species and its related species, it is confirmed as a new species of *Aristolochia* which has strongly curved perianth, 3-lobed limb, as well as 3-lobed gynostemium and should be assigned to the subgenus Siphisia. The new species presented here was also collected by a famous Chinese botanist, Professor K. M. Feng in 1959 (KUN, No. 0163232) from northwest Yunnan, China. The specimen consists of four leaves, being complemented by good field notes and was identified as *Aristolochia hainanensis*. In this paper, this new *Aristolochia* species is described and illustrated.

## Material and methods

Measurements and morphological character assessments of the possible new species *Aristolochia sinoburmanica* were made from both dried specimens and field observations of living plants which allowed comparison of morphological characters and colouration of the perianth (utricle, tube and limb) as well as morphology of the inside of the trap flowers, including the gynostemium, which are often impossible to observe in dried specimens. The description of the new species follows the terminology used by Hwang et al. (2003) and Do et al. (2015a). This new species was compared with the morphologically similar species *A. hainanensis* Merrill, *A. saccata* Wallich and also the recently published new species *A. xuanlienensis* (Huong et al. 2014), *A. faviogonzalezii* T. V. Do, S. Wanke & C. Neinhuis and *A. tonkinensis* T.V. Do & S. Wanke from Vietnam (Do et al.2015a), according to the descriptions from type specimens and dried herbarium specimens and also literature descriptions (Hwang1988, Hwang et al. 2003, Huong et al. 2014, Ma 1989, Do et al. 2015a, 2015b). Protologues and images of type specimens and dried herbarium specimens were gathered from JSTOR Global Plants (http://plants.jstor.org) and the KUN website (http://db.kun.ac.cn/).

## Data resources

The data underpinning the analyses reported in this paper are deposited in the Dryad Data Repository at https://doi.org/10.5061/dryad.2501p.

## Taxonomy

### Aristolochia sinoburmanica

Taxon classificationPlantaeORDOFAMILIA

Y.H.Tan & B.Yang sp. nov.

urn:lsid:ipni.org:names:60475913-2

Figures 1 , 2

#### Diagnosis.

*Aristolochia sinoburmanica* is morphologically similar to *A. hainanensis* Merrill, *A. saccata* Wallich, *A. xuanlienensis* (Huong et al. 2014), *A. faviogonzalezii* T. V. Do, S. Wanke & C. Neinhuis and *A. tonkinensis* T.V. Do & S. Wanke from Vietnam (Do et al. 2015a), but is distinguishable from these species by the following diagnostic characters: leaf blade ovate or ovate-lanceolate to narrowly ovate, subcoriaceous, base rounded to slightly cordate; cyme solitary on old woody stems and young branches, each cyme with 1–2 flowers; perianth claret (deep purple red), outside densely brown hirsute with parallel dark purple veins, 6.5–7.5 cm high; tube horseshoe-shaped, 8.3–8.5 cm, uniformly claret (deep purple red), with visible dark purple veins, limb trumpet-shaped, 4.2–4.8 cm high, 4–4.4 cm wide, 3-lobed, lobes subequal; throat deep purple red, glabrous. The summary and main characters comparison is presented in Table 1.

**Table 1.** Morphological comparison of key characters and distribution in *A. sinoburmanica* and its similar taxa.

Character	*A. sinoburmanica*	*A. faviogonzalezii*	*A. hainanensis*	*A. tonkinensis*	*A. saccata*	*A. xuanlienensis*
Leaf blade	ovate or ovate-lanceolate to narrowly ovate	broadly ovate to cordate	ovate to ovate-lanceolate	ovate to broadly-ovate	ovate-oblong to ovate-lanceolate	ovate or lanceolate-ovate to narrowly ovate
Leaf base	rounded to slightly cordate	slightly to deeply cordate	cuneate to rounded	rounded to truncate	cordate	rounded
Inflorescence	cyme solitary on old woody stems and young branches, each cyme with1–2 flowers	cluster of 6–8(-10) cymes at each node on old woody stem, each cyme with 3–4 flowers	cyme in axils of leafy shoots or on old woody stems, with 3–6 flowers	cyme solitary on old woody stems and young branches with 3–4 flowers	cluster of 2–3 cymes at each node on old woody stems, each cyme with 3–5 flowers	cyme usually in axils of leafy shoots or on old woody stems, 3–4(-5) flowers
Perianth	claret (deep purple red), outside densely brown hirsute with parallel dark purple veins, 6.5–7.5 cm high	yellowish-white with parallel dark purple veins or dots, 3.5–5 cm high	yellowish with obscure purplish veins	outside white, densely villous with parallel purple veins, 3.2–3.5 cm high	white with purple reticulate veins	white with light purple veins outside and dark purple spots, 4.5–4.8 cm high
Limb	discoid-rotund or trumpet-shaped , 4.2–4.8 cm high, 4–4.4 cm wide, 3-lobed, lobes subequal, dark purple, densely covered with dark purple warts	trumpet-shaped, nearly rectangular, 2.4–2.6 cm high, 1.8–2 cm wide, 3-lobed, lobes unequal, dark purple, warty on inner surface	obliquely trumpet-shaped, nearly circular, 2.2–2.5 cm wide, 3-lobed, lobes unequal, purple, densely dark purple warts	trumpet-shaped, nearly rectangular, 1.2–1.3 cm high, 0.9–1.0 cm wide, 3-lobed, lobes unequal, dark purple, densely covered with dark purple bristles	obliquely trumpet-shaped, nearly circular, 1.8–2 cm wide, 3-lobed, lobes unequal, upper 2 distinctly recurved, deltoid, lower one broadly deltoid, covered with purple warts	trumpet-shaped, ca. 2.5 cm wide, 3-lobed , lobes subequal , fused with margins of all lobes strongly revolute, densely covered with purple papillate
Throat	deep purple red, glabrous	upper half white with dark purple dots and lower half pinkish without visible dots	yellow, without visible dots	white, without visible dots	yellow, without visible dots	white, densely covered with purple dots
Distribution	China, Myanmar	Vietnam	China, Vietnam	Vietnam	China, Bhutan, NE India, Myanmar, Nepal	Southern Vietnam

#### Type.

MYANMAR. Kachin State: Putao, near Shinshanku, on the roadside slope of a mountain range bordering the zone of Hkakaborazi National Park, perennial lianas under tropical mountain broadleaf forest, 27°38'48.65"N, 97°54'01.61"E, 900 m a.s.l., 11 May 2017, *Myanmar Exped. 1532* (holotype HITBC!).

**Figure 1.**
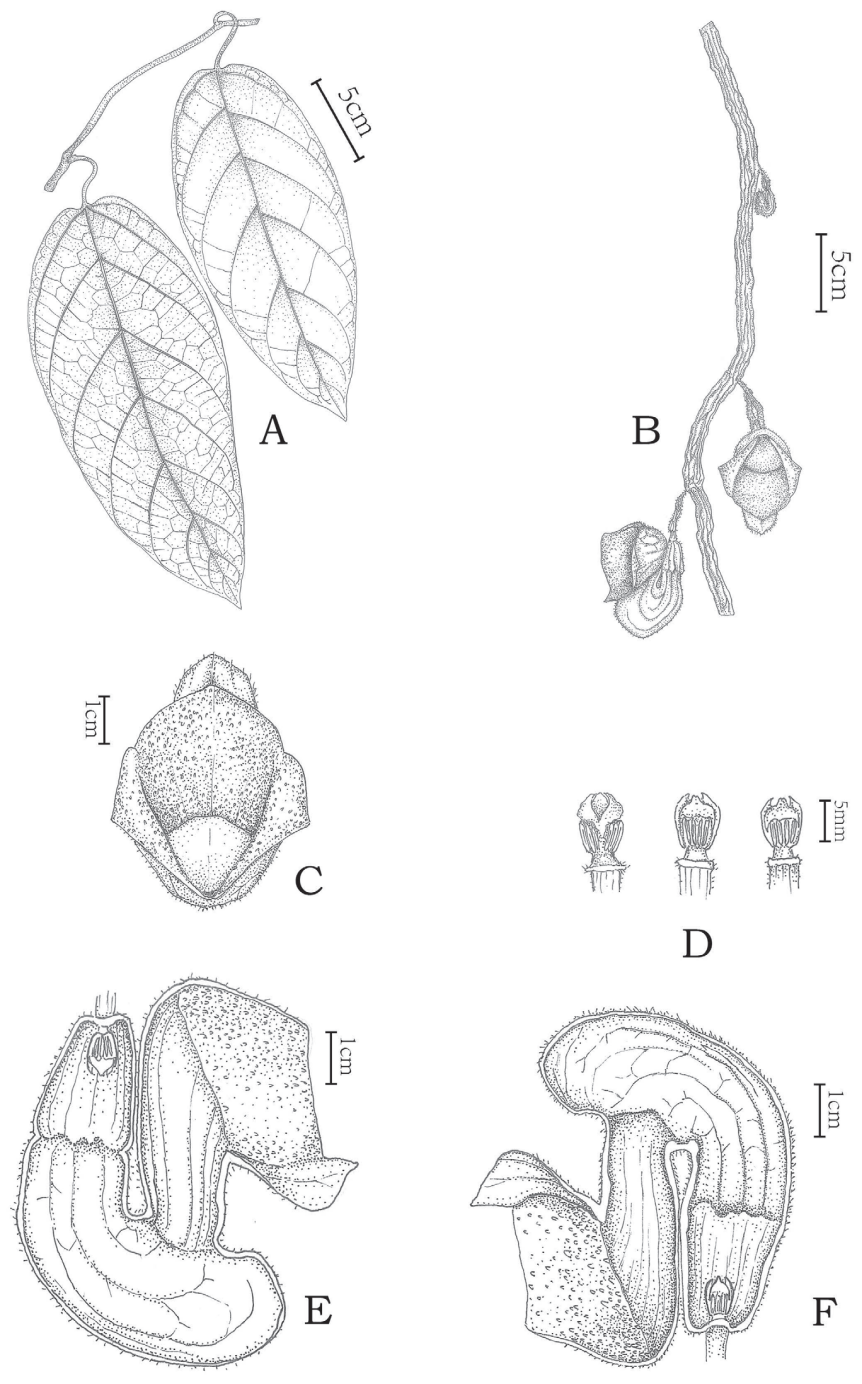
*Aristolochia sinoburmanica* Y.H.Tan & B.Yang, sp. nov. **A** Habitat **B** Flowering branch **C** Flower (front view) **D** Anthers and gynostemium **E** Opened flower (showing the inside structure) **F** Opened flower (showing the inside structure). Illustration by Zhengmeng Yang.

#### Description.

Perennial woody liana, 8–12 m high. Stem terete, circular in cross section, ca. 1 cm in diam., young branches green, sparsely pubescent, becoming glabrescent, bark deeply irregularly longitudinally fissured when mature, internodes 7–15 cm long. Petiole 3.5–6.0 cm long, twisted, pubescent. Leaf blade ovate or ovate-lanceolate to narrowly ovate, subcoriaceous, 15–31.5 × 5.8–12.5 cm, base rounded to slightly cordate, apex acuminate, margin entire, both surfaces densely villous when young, then the adaxial surface dark-green and glabrous, the abaxial surface densely villous; basal veins five, palmate, secondary veins four to six pairs, pinnate; tertiary veins coarsely reticulate, slightly sunken adaxially, prominent abaxially. Inflorescence cymose on old woody stems and young branches, solitary or two cymes, each cyme with one or two flowers, clearly separated from each other. Inflorescence axis 2–6 mm long, tomentose. Bracteole clasping the axis, ovate-triangular, 2–4.5 × 1.5–2 mm, subsessile, both surfaces densely brown villous. Pedicel 1.3–1.6 cm long, pendulous, densely brown villous. Ovary oblong, 1.5–1.7 × 0.3–0.4 cm, densely brown villous. Perianth horseshoe-shaped (in lateral view), 6.5–7.5 cm high, claret (deep purple red), outside densely brown hirsute with parallel dark purple veins, inside glabrous to white villous. Utricle distinct from the tube, bell-shaped, 2.3–2.5 cm high, 1.2–1.3 cm in diam. at base, 1.6–1.8 cm in diam. at apex, inside vinaceous (purplish red) with densely white trichomes. Tube horseshoe-shaped, 8.3–8.5 cm, uniformly claret (deep purple red), with visible dark purple veins, lower tube strongly inflated, saccate, 2.5–2.7 cm high, 1.8–2.0 cm in diam. and upper tube obliquely shortened funnel-shaped, parallel to the utricle, inner surface stramineous dyed with purple red patches, 3–3.5 cm high, narrower at base, 1–1.1 cm in diam. and broader at apex, 1.3–1.4 cm in diam. Limb discoid-rotund or trumpet-shaped, 4.2–4.8 cm high, 4–4.4 cm wide, with three subequal lobes, valvate preanthesis, lobes broadly ovate-triangular, each 3.3–3.8 cm wide, 1.8–2.2 cm high, margins of all lobes revolute during anthesis, warty on inner surface of lobes dark purple. Throat deep purple red, glabrous, without dots. Annulus present. Gynostemium with trilobed stigma on top, 7–7.5 × 5.5–6.5 mm in diam., anthers 6 in 3 pairs, oblong, 4.3–4.5 × 1.2–1.4 mm, yellow. Fruit and seeds were not seen.

#### Phenology.

Flowering specimens have been collected in May but it is possible that flowering begins in April and fruiting may be from July to August.

#### Etymology.

The species epithet refers to the type locality in Myanmar and adjacent regions of China. It also shows that the two countries are friendly neighbours, their friendship being retained over a long period and also expresses our appreciation for the whole-hearted cooperation amongst members of the China-Myanmar joint expedition.

#### Distribution and habitat.

*Aristolochia sinoburmanica* is hitherto known from the type locality of Putao, Kachin state in north Myanmar and adjacent regions of Gongshan County, northwest Yunnan, southwest China, where, according to one sheet of the specimen deposited in KUN, it is a perennial liana which grows under the montane broadleaf forests, at an elevation of ca. 900–1400 m.

#### Preliminary conservation status.

In Nov. 2014, the China-Myanmar joint expedition conducted the first field investigation of plant diversity along the same route in north Myanmar, within the area which included the type locality of this new species. The path through the mountains could only be accessed by foot, but in the most recent expedition in May 2017, with the development of road construction, most of the trees and habitats have been destroyed. *A. sinoburmanica* is known from a single population on the roadside. In fact, during the present study, only two healthy individuals were located growing about 20m apart from each other. Therefore, the new species is assigned a preliminary status of vulnerable (VU) according to the IUCN Red List Categories (IUCN 2012). However, since very few details exist about its natural distribution, a detailed investigation of the same habitats may identify more populations and individuals of this new species. The lack of sufficient data currently does not allow a final risk evaluation and the species might be regarded as data deficient (DD). Further field surveys in northern Myanmar are needed to gain more information on its distribution.

**Figure 2.**
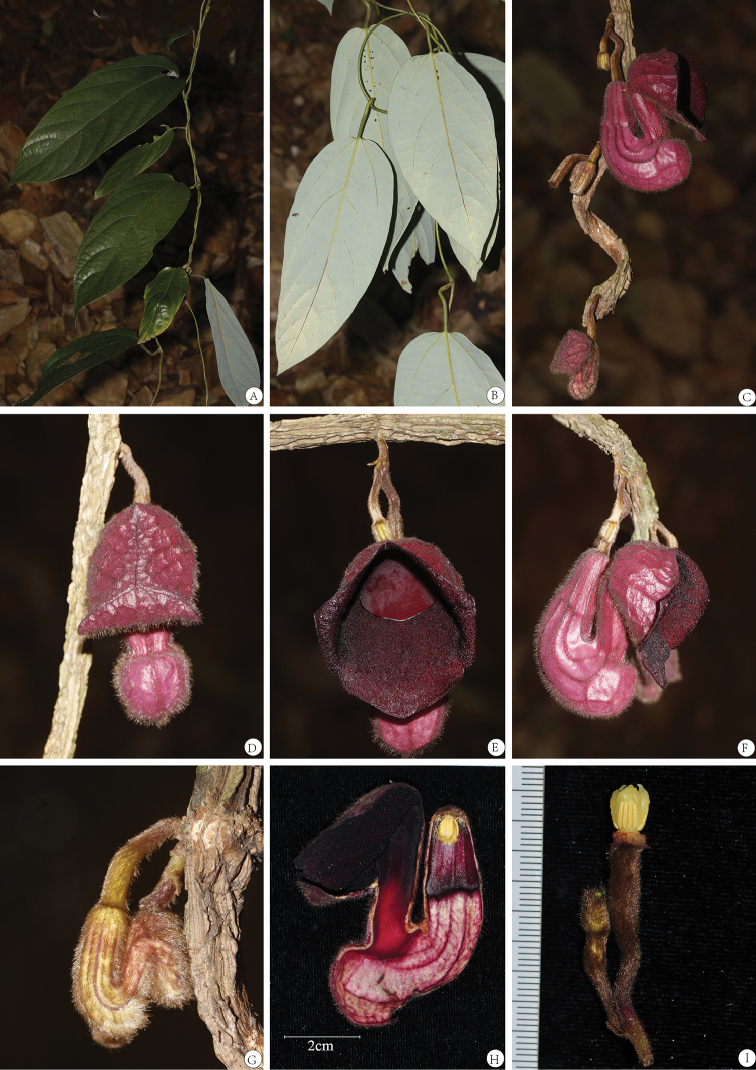
*Aristolochia sinoburmanica* Y.H.Tan & B.Yang, sp. nov. **A** young branch and adaxial leaf **B** young branch and abaxial leaf **C** cymes on old woody stems **D** front view of preanthesis flower **E** front view of open flower **F** lateral view of open flower **G** lateral view of young flower **H** longitudinal section of flower **I** gynostemium, ovary and pedicel. (Photographed by Y. H. Tan, H. B. Ding & B. Yang).

#### Additional specimens examined.

China. Yunnan: Gongshan, east of Dulong River, 27°41'51.81"N, 98°19'11.22"E, 1400 m a.s.l., 12 Nov. 1959, *G.M. Feng. 24217* (KUN, No. 0163232).

#### Key to the species of *Aristolochia sinoburmanica* and closely related species

**Table:** 

1	Perianth claret (deep purple red), outside densely brown hirsute with parallel dark purple veins	***A. sinoburmanica***
–	Perianth yellowish, yellowish-white or white with purple to dark purple veins or dots	**2**
2	Leaf base slightly to deeply cordate	**3**
–	Leaf base round, cuneate to rounded or rounded to truncate	**4**
3	Leaf blade broadly ovate to cordate	***A. faviogonzalezii***
–	Leaf blade ovate-oblong to ovate-lanceolate	***A. saccata***
4	Throat yellow	***A. hainanensis***
–	Throat white	**5**
5	Limb 3-lobed, lobes unequal, throat without visible dots	***A. tonkinensis***
–	Limb 3-lobed, lobes subequal, throat densely covered with purple dots	***A. xuanlienensis***

## Discussion

*Aristolochia sinoburmanica* is morphologically similar to *A. faviogonzalezii*, *A. hainanensis*, *A. tonkinensis*, *A. saccata* and *A. xuanlienensis*. However, the new species differs from the aforementioned species in several important vegetative and reproductive characters (summarised in Table 1). *A. sinoburmanica*, with a horseshoe-shaped perianth of 3 lobes which are valvate in preanthesis, annulated perianth throat and gynostemium with trilobed stigma on top, each lobe consisting of one pair of stamens, belongs to the Aristolochia subgenus Siphisia (Wanke et al. 2006, Do et al. 2015a).This new discovery, along with several new species recently described from Vietnam (Huong et al. 2014, Do et al. 2014, 2015a, 2015b), Guangxi and Hainan Island, China (Xu et al. 2011, Huang et al. 2013, Wu et al. 2013) and Peninsular Malaysia (Yao 2012), provide evidence that the genus *Aristolochia* and, in particular, Aristolochia subgenus Siphisia is very diverse in South-East Asia. Currently there are only 12 *Aristolochia* species recorded in Myanmar (Kress et al. 2003), indicating that the species diversity of *Aristolochia* in Myanmar is still open to discovery. It is predicted that more new species will be discovered when more field investigations are conducted in this region.

## Supplementary Material

XML Treatment for Aristolochia sinoburmanica
